# CLARITE Facilitates the Quality Control and Analysis Process for EWAS of Metabolic-Related Traits

**DOI:** 10.3389/fgene.2019.01240

**Published:** 2019-12-18

**Authors:** Anastasia M. Lucas, Nicole E. Palmiero, John McGuigan, Kristin Passero, Jiayan Zhou, Deven Orie, Marylyn D. Ritchie, Molly A. Hall

**Affiliations:** ^1^Department of Genetics, Institute for Biomedical Informatics, University of Pennsylvania, Philadelphia, PA, United States; ^2^Department of Veterinary and Biomedical Sciences, College of Agricultural Sciences, The Pennsylvania State University, University Park, PA, United States; ^3^Huck Institutes of the Life Sciences, The Pennsylvania State University, University Park, PA, United States

**Keywords:** exposome, quality control, complex traits, metabolic disease, body mass index

## Abstract

While genome-wide association studies are an established method of identifying genetic variants associated with disease, environment-wide association studies (EWAS) highlight the contribution of nongenetic components to complex phenotypes. However, the lack of high-throughput quality control (QC) pipelines for EWAS data lends itself to analysis plans where the data are cleaned after a first-pass analysis, which can lead to bias, or are cleaned manually, which is arduous and susceptible to user error. We offer a novel software, CLeaning to Analysis: Reproducibility-based Interface for Traits and Exposures (CLARITE), as a tool to efficiently clean environmental data, perform regression analysis, and visualize results on a single platform through user-guided automation. It exists as both an R package and a Python package. Though CLARITE focuses on EWAS, it is intended to also improve the QC process for phenotypes and clinical lab measures for a variety of downstream analyses, including phenome-wide association studies and gene-environment interaction studies. With the goal of demonstrating the utility of CLARITE, we performed a novel EWAS in the National Health and Nutrition Examination Survey (NHANES) (N overall Discovery=9063, N overall Replication=9874) for body mass index (BMI) and over 300 environment variables post-QC, adjusting for sex, age, race, socioeconomic status, and survey year. The analysis used survey weights along with cluster and strata information in order to account for the complex survey design. Sixteen BMI results replicated at a Bonferroni corrected p < 0.05. The top replicating results were serum levels of g-tocopherol (vitamin E) (Discovery Bonferroni p: 8.67x10^-12^, Replication Bonferroni p: 2.70x10^-9^) and iron (Discovery Bonferroni p: 1.09x10^-8^, Replication Bonferroni p: 1.73x10^-10^). Results of this EWAS are important to consider for metabolic trait analysis, as BMI is tightly associated with these phenotypes. As such, exposures predictive of BMI may be useful for covariate and/or interaction assessment of metabolic-related traits. CLARITE allows improved data quality for EWAS, gene-environment interactions, and phenome-wide association studies by establishing a high-throughput quality control infrastructure. Thus, CLARITE is recommended for studying the environmental factors underlying complex disease.

## Introduction

Genome-wide association studies (GWAS) have been successful at identifying variants associated with complex disease; yet, it is becoming clearer that many complex diseases have environmental contributions as well (Dempfle et al., 2008). Environment-wide association studies (EWAS) have identified behaviors and exposures that are associated with a given phenotype, such as with type II diabetes (Patel et al., 2010; Hall et al., 2014), high blood pressure (McGinnis et al., 2016), metabolic syndrome (Lind et al., 2013), and arterial disease (Zhuang et al., 2018). Though the backbone of EWAS is regression, a traditional and established statistical method, the series of data preprocessing steps the user takes to reach the analysis stage has not been standardized. Where GWAS shines is its standardized genomic quality control (QC) pipelines (Lemke et al., 2010; Turner et al., 2011; Ellingson and Fardo, 2016; MacArthur et al., 2017), made easily executable by a variety of popular and sophisticated platforms, such as PLINK (Purcell et al., 2007), PLATO (Hall et al., 2017; Ritchie Lab and Geisginger Health Systems, 2017), and GCTA (Yang et al., 2011). EWAS, however, falls behind GWAS in this regard. In contrast to single nucleotide polymorphism (SNP) and other types of genetic data, the majority of environmental data, such as survey data or clinical lab measures, are often cleaned manually. The lack of standardization of high-throughput pipelines for these types of data lends itself to analysis plans where the data are not cleaned until after a first-pass analysis is initially completed, and this can lead to false negatives (**Supplemental Figures 1** and **2**, **Supplemental Table 1**) and/or data-fudging (Van Den Broeck et al., 2005; Motulsky, 2015; Peng, 2015). Alternately, data are cleaned manually before analysis, which often takes great time and effort. Furthermore, manual cleaning is subjective since it relies on the individual investigator’s assessment of the data, leading to a “cleaned” dataset having potentially many different forms. This subjective nature of data cleaning contributes to the reproducibility problems that have plagued the field and have been the source of increasing criticism (Gentleman et al., 2004). Still, removing the user completely from the data cleaning process is both unwise and infeasible. We recognize that it is in the best interest of the individual study to treat each dataset differently depending on both the data itself and the inferences made. Here, we propose an easily accessible software package (available in both R and Python), CLeaning to Analysis: Reproducibility-based Interface for Traits and Exposures (CLARITE), as a tool to ease the process of cleaning environmental and trait data through user-guided automation.

The goal of CLARITE is to guide a dataset from the “raw” data stage to EWAS analysis and subsequent visualization of results. The package is designed to lead a user through the stages of data cleaning: from generating descriptive statistics, to making QC decisions informed by the descriptive statistics, to running analyses on the filtered dataset and visualizing the results. CLARITE’s framework consists of a number of functions intended for filtering, summarizing, and plotting continuous, categorical, and binary data. We recognize that users may have different needs that fall outside of our package. For this reason, the code for the CLARITE R package is publicly available on GitHub (https://github.com/HallLab/clarite) and can be easily modified. The same is true of the Python package (https://github.com/HallLab/clarite-python) which also has online documentation (https://halllab.github.io/clarite-python/) and is available on the Python Package Index (https://pypi.org/). For simplicity, the analysis is described here using the R package, although it was performed using both versions with concordant results.

Herein, we introduce CLARITE as a high-throughput method of cleaning and analyzing environmental data. To demonstrate this, we applied CLARITE to a novel EWAS of body mass index (BMI) using data from the National Health and Nutrition Examination Survey (NHANES), obtained from https://github.com/chiragjp/nhanes_scidata (Patel et al., 2016; Kohane and Avilach, 2018), which contains over 1,100 measures on a total of 22,624 adult samples across four surveys from 1999–2006. Here, we present our QC pipeline and EWAS on categorical, binary, and continuous exposures (including questionnaire, nutrient, and pharmaceutical measures, among others) on BMI in Discovery and Replication datasets. Results of this EWAS are important to consider for metabolic trait analysis, as BMI is tightly associated with these phenotypes, and as such, exposures predictive of BMI may be useful for covariate and/or interaction assessment of metabolic-related traits.

By creating a robust pipeline from the raw data stage through EWAS, we show that cleaning and analyzing a dataset of this size can be streamlined and is feasible from both a user and computational standpoint. The pipeline described here using CLARITE is recommended for ensuring high quality data included in big data analyses that utilize large-scale environmental and/or phenotype data such as EWAS, phenome-wide association studies (PheWAS), and gene-environment interaction analysis. Improving the quality of data with CLARITE will allow for increased reproducibility and replication of results and ultimately will lead to enhanced understanding of the environmental underpinnings of complex human phenotypes.

## Methods

### NHANES Dataset

The focus of this study was to identify associations between BMI and environmental exposures in adults using data from the NHANES, a program aimed at studying health and nutrition for adults and children in the United States. The survey consists of dietary, socioeconomic, and general health measurements obtained through both questionnaire responses and clinical laboratory measurements. Curated data for 41,474 individuals and 1,191 variables across four survey periods (1999–2000, 2001–2002, 2003–2004, 2005–2006), are freely available on GitHub (https://github.com/chiragjp/nhanes_scidata) and through the NHANES Dataset Explorer (Patel et al., 2016; Kohane and Avilach, 2018).

Participants included in the present study were at least eighteen years of age at the time of study participation. We employed a discovery and replication protocol in our pipeline, whereby QC and EWAS were first performed in a specified Discovery set of the NHANES data and the results that were significant when allowing for a 10% false discovery rate (FDR) were considered for replication in a separate subset of NHANES (Replication dataset). Participants in the 1999–2000 and 2001–2002 NHANES data release cycles were included in the Discovery dataset (N = 9,063; 52% female, 47% white, 19% black, 25% Mexican, 5% other Hispanic, 3% other ethnicity) and participants in the 2003–2004 and 2005–2006 NHANES data release cycles were included in the Replication dataset (N = 9,874; 52% female, 49% white, 23% black, 21% Mexican, 3% other Hispanic, 1% other ethnicity). For both Discovery and Replication datasets, only variables available for both survey years within the datasets were considered for analysis. For instance, if an exposure was only available in release cycle 1999–2000, it was dropped and not considered for Discovery EWAS. Additionally, variables were excluded if a suitable survey weight could not be determined based on the NHANES documentation (https://wwwn.cdc.gov/nchs/nhanes/ContinuousNhanes/).

### Accounting for the Complex Sampling Design of NHANES

Complex survey designs incorporate survey (sample) weights to ensure sample data is representative of the population from which it was obtained (Lumley, 2004; Johnson et al., 2013). Sample weights both account for the probability of selecting a given individual for a survey and adjust the contribution of each sample in the analysis to correct under- or over-representation of population subgroups (Lee and Forthofer, 2006). Data collected using a complex design can lead to biased results if the data is treated as though obtained through simple random sampling (Lumley, 2004; Lee and Forthofer, 2006). Incorporating survey weights and accounting for the complex survey design is prudent for unbiased analysis of NHANES data.

CLARITE was used along with the R *survey* package to account for the strata, primary sampling units (PSU), and sample weights available in the NHANES data. Variance was estimated using the Taylor series linearization method (Lumley, 2004). CLARITE’s *ewas()* function accepts survey weights and utilizes the correct variance estimation procedure to account for PSU and strata. It can accept a single weight to use for all tests, or for heterogeneous tests, where the desired weights vary depending on the variables and covariates in the model, CLARITE can flexibly accept a named list of weights that match each regressed variable with the correct weight for that model. As per the NHANES guidelines, each variable was matched with the weight corresponding to the smallest subsample of individuals (Johnson et al., 2013).

The NHANES data collection for the 1999–2006 surveys was tiered. All samples were included in the home interview stage, from which a random sample was selected to participate in the mobile examination center (MEC) assessment. From the MEC subsample, smaller random subsamples were derived to collect additional information (ex: dietary recall subsample, fasting subsample, laboratory environmental subsample). For each survey cycle, distinct 4-year/2-year weights were calculated for the interview section, MEC examination, and individual subsamples. Each regression model retained the outcome, *logBMI* (calculated from the variable *BMXBMI*) and the covariates (sex, ethnicity, socioeconomic status, age, and survey year). Each covariate was assessed in the home interview portion of the survey and was thus assigned the home interview weight. *BMXBMI* was measured in the MEC examination and assigned MEC weights. Because the MEC examination comprised a smaller subsample than the home interview, the MEC weights were used for each regression model unless the predictor variable came from a smaller analytic subsample. When low-density lipoprotein (LDL) was assessed as a potential confounder, the weight for the LDL measure was used because it was measured in a smaller subsample than the predictor variables. The variables from the Unified NHANES dataset were manually assigned their respective four-year and/or two-year weights.

Prior to 2000, a 2-year survey weight was calculated for each survey cycle using data from the 1990 U.S. Census. Beginning with the 2001–2002 data release, the sample weights were calculated using data from the new 2000 U.S. Census. Four-year survey weights are available in NHANES for combined analysis of these two survey cycles. For the replication analysis, the NHANES guidelines for creating a combined 4-year survey weight were followed.

### Data Quality Control Using CLARITE in Discovery Dataset

**Figure 1:**
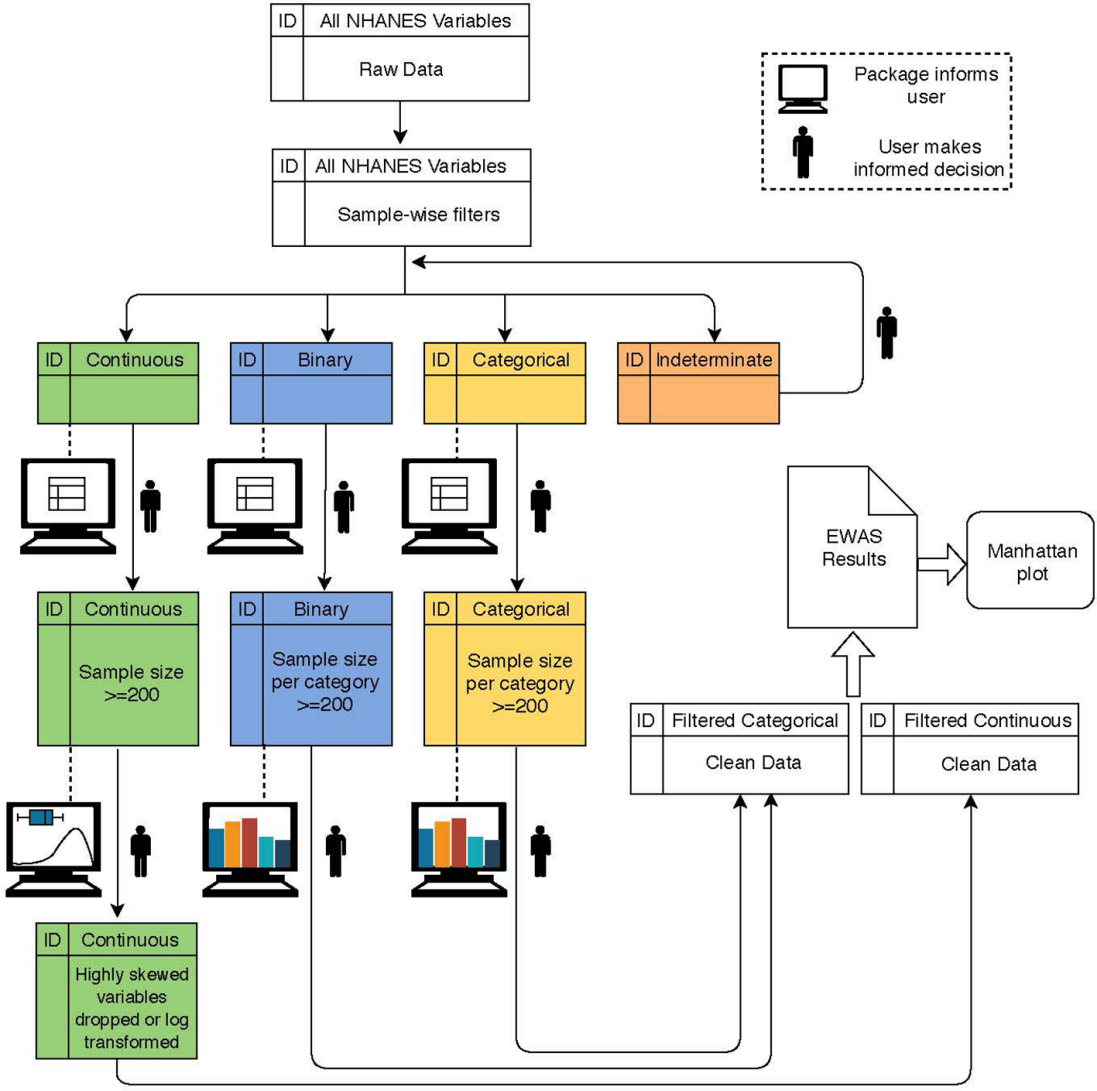
Flowchart depicting a typical workflow when using the CLARITE package. The user starts with raw data and alternates between summary steps (dashed lines) and filtering/quality control (QC) steps (solid lines) based on variable type (indicated by color) and either user-defined or default thresholds informed by the summary output. Once data are sufficiently cleaned, environment-wide association studies (EWAS) can be run.

A broad overview of CLARITE’s QC, analysis, and visualization pipeline is displayed in **Figure 1**. In order to ensure data integrity, the following QC pipeline was executed using the CLARITE R package. Prior to QC, 962 environmental variables from the NHANES dataset were considered for the Discovery dataset.

#### Missingness-Based Sample Exclusion

Of the initial Discovery participants in the NHANES dataset, 9,063 had nonmissing values for BMI and all covariates (age, sex, socio-economic status (SES), self-reported race, and data release cycle year) and were included for analysis. Any participant with a missing covariate, survey weight, or BMI value was excluded.

#### Variable-Type Extraction

The NHANES dataset contains mixed variable types (i.e., binary, categorical, and continuous variables). To split the data into sets of homogenous variables, we used CLARITE’s collection of get* functions, *get_binary()*, *get_categorical()*, *get_continuous()*, and *get_check()*, the last of which returns variables that the package is not able to confidently identify and the user will need to sort by hand. These functions work by extracting variables according to a user-defined minimum and maximum number of unique values in each variable (note: *get_binary()* always extracts variables with two unique values and does not take a min/max user input). For this study, variables having between 3 and 5 unique values were extracted as categorical (*get_categorical()*) and those with 15 or more unique values were extracted as continuous (*get_continuous()*). Those variables with between 6 and 14 unique values were initially extracted as indeterminate/ambiguous (*get_check()*) for manual inspection and then added to the continuous or categorical lists accordingly using CLARITE. The variable-type extraction step is critical to downstream QC and analysis, as different filters are only appropriate for certain variable types. This family of functions is one of CLARITE’s premier features as classifying variables by data type can be one of the most important and time-consuming QC steps. The get* functions reduced the number of variables that needed to be manually sorted from >900 variables in our initial dataset to just 22 that were flagged as indeterminate by CLARITE.

#### Sample-Size-Based Variable Exclusion

To ensure optimal power, variables with sample size less than 200 were dropped as recommended previously (Wilson Vanvoorhis and Morgan, 2007). For categorical/binary variables, a further requirement of at least 200 samples for every category was implemented.

#### Data Distribution Check

CLARITE also has the ability to perform high-throughput data visualization on continuous measures (histograms, quantile-quantile plots, box plots) and categorical/binary variables (bar charts). Histograms were generated to visually inspect the distributions of the continuous variables for abnormalities. The distribution of BMI was visibly right skewed; thus, a log transformation was performed on the BMI values to normalize the data and address the skewness (**Supplemental Figures 3A, B**). Visualization of continuous exposure variable histograms further allowed identification of several variables with some samples having a highly skewed distribution. For the majority of these cases, the variable was assessing use of a supplement (e.g., calcium supplement). To restrict our analysis to variables with sufficient nonzero values, we removed any variable that had more than 90% of the samples with a value of zero. As seen in **Table 1**, Discovery QC left 376 remaining exposures for EWAS analysis: 312 continuous, 60 binary, and 4 categorical variables. Replication QC using the same procedure left 419 exposures (343 continuous, 71 binary, and 5 categorical). Only variables passing QC in both datasets were retained for the EWAS portion of the analysis, leaving the same 332 exposures (280 continuous, 48 binary, and 4 categorical) in each dataset.

**Table 1:** Environmental variables and sample sizes for environment-wide association studies (EWAS) in Discovery and Replication datasets.

Dataset	Binary	Categorical	Continuous	Overall Sample Size
Discovery	60	4	312	9063
Replication	71	5	343	9874
Shared	48	4	280	n/a

Number of variables remaining and overall sample size after quality control (QC) included for EWAS in the Discovery and Replication datasets.

#### Additional Features of CLARITE

Although they are not mentioned in the QC steps above, CLARITE also provides several additional commonly used functions for data management and data exploration. On the data management side, CLARITE has functions to quickly merge data frames and filter data by variables and samples. Further, due to the common issue of analyzing data with multiple missing values or different missing values across variables, we have included a function which standardizes the missing values based on a user provided data map file. For the purpose of data exploration, several summary statistic-based functions are also available. Users have the ability to create frequency tables for their categorical or binary data and obtain Pearson correlation coefficients for their continuous data. Additionally, for continuous measures, we have made it easier to evaluate the impact of outliers prior to filtering by including a function that will compare the summary statistics of a dataset before and after removing outliers at a user-specified standard deviation.

#### Environment-Wide Association Study (EWAS) Using CLARITE in Discovery Dataset

Using CLARITE, EWAS was run using linear regression models for each of the 332 variables that passed QC in both datasets, adjusting for sex, self-reported race (white, black, Mexican, other Hispanic, or other ethnicity), socioeconomic status (SES), age, and survey year. CLARITE’s *ewas()* function uses R’s generalized linear model (glm) function, and as such, will report the same betas and p-values as would ordinarily be reported by *glm()* in a typical regression analysis. When complex survey data is provided, the strata, cluster, and weight information is passed to the *survey* R package. In the case of continuous predictors, the beta and p-value of the variable in the model are reported in CLARITE’s *ewas()* as in R’s *glm()*; however, in the case of categorical predictors, R reports a separate beta and p-value for each level/category of the variable. In order to assess the overall impact of the categorical variable, CLARITE performs a likelihood ratio test (LRT) between the full (including predictor of interest) and reduced models (excluding the predictor of interest) using R’s *anova()* function and therefore reports a single LRT p-value for the variable. The regression models for full and reduced models for categorical predictors can be written as *Y* = *β*_0_ + *β*_1_*X* + *β*_2_*cov*_1_ + … + *β_n_*_+1_*cov_n_* and *Y* = *β*_0_ + *β*_1_*cov*_1_ +…+ *β_n_cov_n_*, respectively, where *X* is again a predictor variable of interest and *Y* is the phenotype. Along with this, it is important to note that when running EWAS on categorical predictors without covariates, CLARITE will consider the reduced model to be the null model.

For each replicating result, further potential confounding metabolic-health related traits were assessed, including: Type II Diabetes (T2D), coronary artery disease (CAD), high-density lipoproteins cholesterol (HDL), low density lipoprotein cholesterol (LDL), total cholesterol (TC), and triglycerides (TG). Each of these potential traits were included as covariates individually with the original covariates.

The *ewas* function takes an optional *min_n* parameter which will prevent a variable from being included in the EWAS if there are fewer than that number of samples with no missing values across all variables for that particular regression model, instead returning a NULL result and printing a warning to the console. All of these analyses were run using the default *min_n* value of 200. Observations with a weight of zero are also discarded, as these observations are ignored when compensating for weights in the regression calculation. All variables run in the primary analyses had sufficient sample sizes, but some dropped below the threshold when adding the metabolic-health traits as covariates. **Table 3** shows NA when this occurs, since no p-values were calculated because sample sizes were below 200.

#### Replication QC and EWAS

To allow the opportunity for replication of results, we selected from the Discovery EWAS those variables associated with BMI with an FDR < 10% (Discovery threshold) for consideration in the Replication EWAS (99 exposures). Replication involved the same protocol and covariate adjustment as the Discovery EWAS, analyzing these 99 variables (85 continuous, 12 binary, and 2 categorical).

Lastly, Manhattan plots were generated using CLARITE’s *eman()* function to display the EWAS results. In EWAS Manhattan plots, users can organize variables into categories along the x-axis, analogous to how SNPs are organized into chromosomes for GWAS, so patterns of significance across categories can be easily observed.

## Results

### Replicating EWAS Results

**Figure 2:**
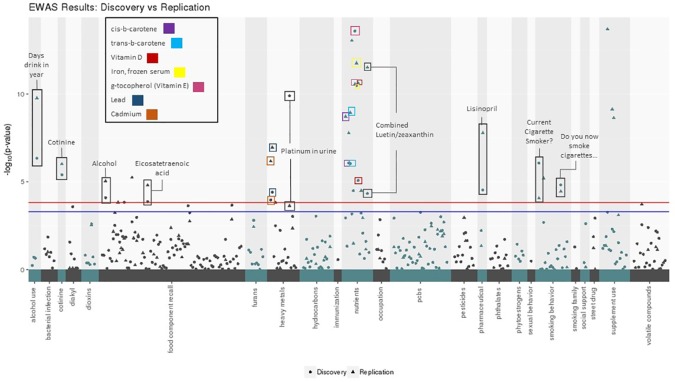
Environment-wide association studies (EWAS) results for body mass index (BMI) in Discovery and Replication datasets using CLARITE. Manhattan plot displays exposure categories along the x- axis with -log10(p-value) along the y-axis, results included for Discovery (circle) and Replication (triangle) datasets. The red line denotes the Bonferroni threshold (alpha: 0.05) for the number of tests run in the Discovery dataset (305), and the blue line denotes the Bonferroni threshold (alpha: 0.05) for the number of tests run in the Replication dataset (99). The 16 replicating results with Bonferroni-corrected p-value < 0.05 are labeled.

Of the 332 exposure variables passing QC in both datasets, 99 were significant in the Discovery dataset when allowing for an FDR < 10%. Of these, 62 were significant in Replication when allowing for an FDR < 10% (**Table 2**). Sixteen EWAS results were significant in both datasets with a Bonferroni corrected p-value below 0.05 and ten passed a Bonferroni corrected alpha of 0.01 in both datasets (based on 332 tests for Discovery and 99 tests for Replication) (**Figure 2**). In addition to replicating significant p-values, these variables demonstrated consistent directions of effect across the Discovery and Replication datasets.

**Table 2:** Overview of number of results in Discovery, Replication, and both datasets at varying significance thresholds.

Dataset	Tests	FDR 0.1	Bonf 0.05	Bonf. 0.01
Discovery	332	99	18	11
Replication	99	62	29	25
Both	NA	62	16	10

Number of tests performed in the discovery and replication analyses, number of results passing false discovery rate (FDR) < 0.1, and number of results passing Bonferroni p-value threshold (alphas 0.05 and 0.01).

The result with the lowest p-value in either Discovery or Replication datasets was g-tocopherol(ug/dl) (vitamin E) (Discovery: unadjusted p: 2.61x10^-14^, Bonferroni p: 8.67x10^-12^; Replication: unadjusted p: 2.73x10^-11^, Bonferroni p: 2.70x10^-9^). The next lowest was iron, frozen serum (ug/dl) (Discovery: unadjusted p: 3.28x10^-11^, Bonferroni p: 1.09x10^-8^; Replication: unadjusted p: 1.75x10^-12^, Bonferroni p: 1.73x10^-10^). Results relating to substance use also showed significance in the both datasets, including smoking behavior (Discovery: unadjusted p: 8.74x10^-7^, Bonferroni p: 2.90x10^-4^; Replication: unadjusted p: 8.63x10^-5^, Bonferroni p: 8.54x10^-3^), measurable amounts of cotinine in the body from smoking (Discovery: unadjusted p: 3.99x10^-6^, Bonferroni p: 1.32x10^-3^; Replication: unadjusted p: 9.67x10^-7^, Bonferroni p: 9.58x10^-5^), and number of days where alcohol was consumed (Discovery: unadjusted p: 4.56x10^-7^, Bonferroni p: 1.51x10^-4^; Replication: unadjusted p: 1.71x10^-10^, Bonferroni p: 1.69x10^-8^). Another top-ranking and replicating result was related to heavy metal exposure: platinum concentration in urine (Discovery: unadjusted p: 1.28x10^-10^, Bonferroni p: 4.25x10^-8^; Replication: unadjusted p: 2.322x10^-4^, Bonferroni p: 0.023).

The ability of CLARITE to organize Manhattan plots using exposure categories allows a global observation of trends in significant associations. Nutrient, food component recall, and heavy metal values contributed the largest number of exposures meeting a Bonferroni corrected significance threshold. In the nutrient category, g-tocopherol demonstrated the lowest p-value, as described above. In the food component recall category, alcohol (gm) demonstrated the lowest p-values (Discovery: unadjusted p: 8.10x10^-5^, Bonferroni p: 0.0269; Replication: unadjusted p: 9.21x10^-6^, Bonferroni p: 9.12x10^-4^). For the heavy metal category, the exposure with the smallest p-values was urine platinum (ug/L), also as described above.

### Evaluation of Replicating Results With Potential Confounding Health-Related Traits

To assess the potential impact of complex metabolic-related phenotypes known to be associated with BMI, the impact of adding each of the following individually as a covariate was evaluated for the 16 replicating exposures that met a Bonferroni corrected p-value threshold of 0.05: type II diabetes (T2D), coronary artery disease (CAD), HDL cholesterol (HDL), LDL cholesterol (LDL), triglycerides (TG), and total cholesterol (TC) (**Supplemental Figures 4A–F**).

Two variables remained Bonferroni significant when adding each of the metabolic-health related traits (alpha: 0.05): g-tocopherol(ug/dl) (vitamin E) and iron (ug/dl) (**Table 3**). All variables except for blood cadmium remained significant in both datasets when adjusting for CAD, and 9 of the 14 remained significant when adjusting for type II diabetes: iron, g-tocopherol, lutein and zeaxanthin, days drink in year, cis-beta carotene, trans-beta carotene, lead, cotinine, “Do you now smoke cigarettes,” “Current cigarette smoker,” and urine platinum.

**Table 3:** Replicating results reaching Bonferroni significance. The first column shows a list of 16 exposures that are Bonferroni significant at the 0.05 level. The fourth and fifth columns are the raw p-values from the original datasets. Columns 6-17 are the Bonferroni corrected p-values from the Discovery and Replication datasets after each adjustment was performed.

Variable	Category	Description	p-val_Dis	p-val_Rep	Bonferroni corrected p-value w/ T2D adjustment (Discovery)	Bonferroni corrected p-value w/ T2D adjustment (Replication)	Bonferroni corrected p-value w/ CAD adjustment (Discovery)	Bonferroni corrected p-value w/ CAD adjustment (Replication)	Bonferroni corrected p-value w/ HDL adjustment (Discovery)	Bonferroni corrected p-value w/ HDL adjustment (Replication)	Bonferroni corrected p-value w/ LDL adjustment (Discovery)	Bonferroni corrected p-value w/ LDL adjustment (Replication)	Bonferroni corrected p-value w/ TC adjustment (Discovery)	Bonferroni corrected p-value w/ TC adjustment (Replication)	Bonferroni corrected p-value w/ TG adjustment (Discovery)	Bonferroni corrected p-value w/ TG adjustment (Replication)
LBXGTC	nutrients	g-Tocopherol (ug/dL)	2.611e-14	2.729e-11	1.147e-10	9.566e-09	4.286e-11	1.031e-08	1.908e-10	1.246e-07	8.675e-07	2.446e-08	2.552e-11	5.372e-09	2.911e-08	6.823e-10
LBXIRN	nutrients	Iron (ug/dL)	3.283e-11	1.748e-12	1.759e-08	4.527e-10	2.185e-08	5.718e-10	7.207e-06	7.294e-08	5.367e-04	3.289e-07	2.178e-08	3.543e-10	5.423e-08	1.001e-09
URXUPT	heavy metals	Platinum, urine (ug/L)	1.281e-10	2.322e-04	1.307e-06	7.970e-02	4.795e-07	2.207e-02	1	NA	1.394e-03	2.342e-04	1.792e-07	2.462e-02	1	NA
total_days_drink_year	alcohol use	days drink in year	4.563e-07	1.710e-10	4.298e-04	3.352e-08	2.426e-04	4.133e-08	1	NA	1	2.329e-06	2.409e-04	2.890e-08	1.973e-04	1.375e-07
LBXBEC	nutrients	trans-Beta carotene (ug/dL)	8.394e-07	1.690e-08	1.996e-03	2.607e-06	1.478e-03	2.657e-06	6.841e-03	1.016e-05	1.719e-01	9.840e-04	1.785e-03	2.363e-06	2.594e-03	4.075e-06
cigarette_smoking	smoking behavior	Current Cigarette Smoker?	8.735e-07	8.627e-05	4.984e-04	1.939e-02	5.460e-04	1.446e-02	8.060e-04	9.956e-04	7.293e-01	1	3.938e-04	1.134e-02	7.821e-04	1.073e-02
LBXCBC	nutrients	cis-Beta carotene (ug/dL)	9.142e-07	1.159e-09	2.233e-03	2.239e-07	1.487e-03	2.177e-07	7.286e-03	9.552e-07	9.814e-02	2.328e-04	1.963e-03	1.835e-07	2.936e-03	2.984e-07
LBXCOT	cotinine	Cotinine (ng/mL)	3.989e-06	9.673e-07	8.159e-04	5.675e-04	1.944e-03	8.101e-05	3.626e-05	1.216e-05	8.630e-02	1.205e-01	1.687e-03	1.264e-04	4.326e-04	4.587e-05
LBXLUZ	nutrients	Lutein and zeaxanthin (ug/dL)	8.463e-06	2.312e-11	1.356e-02	2.214e-09	1.404e-02	7.595e-09	7.692e-02	9.480e-06	4.178e-02	2.035e-09	1.292e-02	4.697e-09	8.614e-03	2.491e-09
SMQ040	smoking behavior	Do you now smoke cigarettes...	1.509e-05	3.521e-05	4.154e-03	6.608e-03	7.607e-03	2.047e-03	1.409e-03	5.894e-04	1	9.251e-01	7.106e-03	4.389e-03	3.412e-03	1.908e-03
LISINOPRIL	pharmaceutical	LISINOPRIL	2.919e-05	1.665e-08	5.157e-01	7.621e-03	6.523e-03	2.183e-06	1.958e-01	7.836e-04	1.209e-02	5.763e-02	1.744e-02	2.942e-06	6.626e-02	3.901e-05
LBXBPB	heavy metals	Lead (ug/dL)	3.858e-05	1.106e-07	1.686e-02	1.037e-04	1.760e-02	1.321e-05	1.411e-02	3.311e-04	2.305e-01	1.160e-04	1.538e-02	1.527e-05	1.811e-02	1.611e-05
LBXVID	nutrients	Vitamin D (ng/mL)	4.627e-05	3.048e-12	6.081e-02	4.580e-09	4.478e-02	2.545e-10	2.821e-01	2.356e-07	1	1.251e-06	4.654e-02	7.294e-10	6.661e-02	2.987e-09
DR1TALCO	food component recall	Alcohol (gm)	8.099e-05	9.210e-06	5.669e-02	2.874e-03	1.474e-02	5.996e-04	1	NA	1	NA	3.821e-02	9.168e-04	3.996e-02	1.217e-03
LBXBCD	heavy metals	Cadmium (ug/L)	1.087e-04	6.826e-07	8.970e-02	3.771e-04	5.373e-02	1.230e-04	8.827e-02	3.398e-05	6.130e-02	9.388e-02	3.916e-02	7.718e-05	3.989e-02	4.872e-05
DR1TP204	food component recall	PFA 20:4 (Eicosatetraenoic) (gm)	1.329e-04	1.573e-05	5.840e-02	2.585e-03	4.481e-02	1.912e-03	1.079e-03	9.831e-04	1.998e-01	5.728e-02	4.506e-02	2.301e-03	4.605e-02	5.319e-03

## Discussion

There has been much concern of late about the problem of reproducibility and replication of results in the sciences (Motulsky, 2015; Peng, 2015). At the heart of poor reproducibility are three main issues: lack of 1) standardized infrastructure (i.e., software to perform QC protocols), 2) clear documentation of QC and analysis protocols, and 3) standardization of QC protocols (Peng, 2015). SNP QC has become standardized in terms of protocols (Laurie et al., 2010; Turner et al., 2011; Zuvich et al., 2011; Verma et al., 2014; Ellingson and Fardo, 2016) and infrastructure (Purcell et al., 2007; Zheng et al., 2012; Hall et al., 2017), and it is commonplace for these to be well-documented in publications employing SNP data. Exposome QC protocol and infrastructure development, on the other hand, have previously received little attention (Engel et al., 2013; Zhu et al., 2013; Emwas et al., 2018). To address this need, we developed CLARITE software as an infrastructure for high-throughput and rigorous QC of exposome data. Employing clear and rigorous QC protocols with clear documentation using CLARITE offers the opportunity for improved reproducibility of results and decreased false positive associations. However, there is still a dearth of research-driven consensus of exposome QC best practices. As more QC protocols are deemed appropriate by the EWAS community and added to CLARITE, the tool’s versatility will grow. We recommend CLARITE to perform high-quality EWAS with exposure big data in order to explore the environmental component of complex traits.

With CLARITE, we explored the environmental etiology of body mass index (BMI), a complex trait with major implications in regard to metabolic disease. The results of this study offer new avenues for increased granularity in associations between BMI and metabolic traits by evaluating the impact environmental factors we identified as covariate adjustment or for interaction analysis. A discovery and replication protocol was employed, whereby QC and EWAS were performed in a Discovery subset of the NHANES. Of the exposures considered, 332 passed QC in both datasets and 99 were significant when allowing for a false discovery rate (FDR) less than 10%. These exposures were considered for replication in a separate Replication subset of NHANES. Of these, 16 exposures were significant with a Bonferroni-corrected p-value less than 0.05 in both Discovery and Replication datasets.

CLARITE was successful at replicating previously known associations. For instance, we identified an association wherein increase in alcohol consumption is associated with increased BMI. Well-established relationships between alcohol consumption and BMI have been discussed in previous research, though the nature of the relationship is complex (French et al., 2010). Another lifestyle factor, smoking exposure, has previously been reported as being associated with BMI (Jain and Bernert, 2010; Dare et al., 2015) and was identified in our Discovery and Replication EWAS for three NHANES variables: *Current cigarette smoker, do you smoke now*, and cotinine. Lead levels demonstrated a replicating positive association with BMI in our EWAS, and this was another previously reported finding (Wang et al., 2015; Dip et al., 2017; Park et al., 2017). Serum iron levels were also found in our NHANES EWAS to replicate in women (measure not available in male participants) with an inverse relationship, an association that has been previously reported (Mujica-Coopman et al., 2015).

The top replicating EWAS result was g-tocopherol (vitamin E) with a positive relationship. One biological explanation for this association is that individuals with elevated BMI have increased systemic and adipose tissue contributing to specific oxidative stress (Kimmons et al., 2006). Adipose or fat tissue was not available in the NHANES datasets to test as a covariate, and therefore, research is needed to further elucidate the relationship between BMI and vitamin E. Of note, despite previously reported associations between vitamin E and lipid levels (Waniek et al., 2017) vitamin E retained significance in our study when adjusting for each lipid covariate, indicating the association between the two measures is relevant even when controlling for lipids. Another top replicating EWAS result was iron, frozen serum (ug/dl), which showed a negative relationship. After applying the adjustments, iron, frozen serum (ug/dl) remained significant.

Due to the influence of BMI on numerous outcomes, we assessed the role of some key metabolic-related health traits in the replicating EWAS results to further evaluate their relationship with BMI. The 16 results with a Bonferroni-corrected p-value less than 0.05 in both Discovery and Replication included blood serum levels of nine different compounds: iron (ug/dl), vitamin D (ng/ml), g-tocopherol (vitamin E, ug/dl), lutein and zeaxanthin (ug/dl), cis-beta carotene (ug/dl), trans-beta carotene (ug/dl), lead (ug/dl), cadmium (ug/dl), and cotinine (ng/mL). They also included the amount of two compounds in a dietary recall survey (alcohol (gm) and eicosatetraenoic acid (gm)), “days drink in year,” “Do you now smoke cigarettes?”, “Current cigarette smoker?”, the amount of platinum in the urine (ug/L), and the use of lisinopril (medication used to treat high blood pressure). All variables except for blood cadmium remained significant in both datasets when adjusting for CAD. Including T2D as a covariate resulted in five variables dropping from Bonferroni significance in the Discovery dataset (vitamin D, Lisinopril, cadmium, and both dietary recall variables), and one in Replication (urine platinum (ug/L)). Only iron and g-tocopherol remained Bonferroni significant in both datasets across all tested potential confounding variables. Exposures that drop from significance shed important information on their relationship with BMI and indicate the exposures’ signals may have been due to covarying associations with metabolic-related traits rather than to BMI itself, reflecting the complex role of correlation structure when evaluating the exposome.

In this study, we demonstrated the utility of CLARITE to rigorously QC environmental big data prior to EWAS. Although we have showcased CLARITE’s ability to improve the QC and analysis pipeline with a use case for EWAS data, this tool is designed to be a general higher-throughput quality control tool that can be applied to many types of data. With this in mind, CLARITE has the potential to provide significant improvements to the QC pipelines for phenotype data and clinical lab measures, particularly in the case of phenome-wide association studies (PheWAS) in which thousands of phenotypes may be assessed at once, and due to the volume of data, QC is often done after first pass analysis, much like in EWAS.

CLARITE does have limitations and future directions include expanding the tool to handle multiple phenotypes and a wider range of statistical functions, including incorporating regression diagnostic functions, such as those from the *gvlma* (Global Validation of Linear Model Assumptions) package (Peña and Slate, 2006) and the Shapiro-Wilk test, to flag possible scenarios where a typical regression would be inappropriate and additional regression methods, such as ordinal and multinomial regression. Other future work will involve more sophisticated analysis options, including, but not limited to, environment-environment interaction analysis as well as the ability to incorporate genetic data for gene-environment analysis. However, the flexibility and open source nature of the tool allow opportunity for users to customize or add functions to fit their analytic and data needs. Finally, it should be noted that NHANES dataset is an example of a fairly well preprocessed dataset. While CLARITE added ease to the QC and analysis process for this EWAS and identified potential problem variables, future applications to less processed datasets, such as electronic health record data, will further showcase CLARITE’s QC abilities.

Effective and efficient quality control is a crucial component of any analysis and should not be ignored due to infeasibility of manual inspection of large datasets. Application of CLARITE to a wide range of environmental and phenotype datasets has the potential to alleviate many of the challenges faced when analyzing large-scale data, while encouraging sound quality control practices as well as reproducibility. This, ultimately, will contribute to uncovering the role of exposures and gene-environment interactions in complex human phenotypes.

## Author Contributions

AL, NP, and MH contributed to conception and design of study. AL, JM and DO developed the package. AL, NP, JM, KP, and JZ performed computational analyses. AL and NP wrote the first draft of the manuscript. All authors contributed to the final draft of the manuscript. MH oversaw and guided the research and manuscript efforts.

## Conflict of Interest

The authors declare that the research was conducted in the absence of any commercial or financial relationships that could be construed as a potential conflict of interest.

The handling editor declared a past co-authorship with several of the authors MR, MH.
