# Supplementary material for: CLARITE Facilitates the Quality Control and Analysis Process for EWAS of Metabolic-Related Traits
Source: Front Genet. 2019 Dec 18;10:1240. doi: 10.3389/fgene.2019.01240 (PMC6930237; doi:10.3389/fgene.2019.01240)
Supplement: Supplementary file 1 [file DataSheet_1.docx]

**SUPPLEMENTARY FIGURES**

**Supplemental Figure 1. Power simulation of linear regression with continuous predictor.** The simulations compared two significance thresholds, the pre-quality-control (pre-QC) Bonferroni significance threshold p < 0.000052 or 0.05/962 and the post-quality-control (post-QC) Bonferroni threshold for our Discovery analysis p < 0.00015 or 0.05/332. The pre-QC threshold indicates the multiple test burden had we not applied an exposome QC protocol. Models were built using pairwise combinations of sample size N = 200 and β-coefficients (from β = 0 to β = 1, by 0.1). Each model was simulated 1000 times and average power was calculated as the number of times the p-value was below the significance threshold. The dashed line indicates 80% power. Average false positive rates where β = 0 are reported in Supplemental Table 1. While sufficiently large effect sizes retained power regardless of the multiple test burden, performing QC to remove low-quality data mitigated the loss of power at lower effect sizes. Since results from association studies tend to have smaller effect sizes (Hall et al., 2016), it is important to adopt practices that recover false negatives across low β-coefficients.

**Supplemental Figure 2. Power simulation of linear regression with binary predictor and varying exposure prevalence.** Simulations used a dichotomous predictor with proportions of exposure as described below. Power was calculated across pre-QC Bonferroni significance threshold p < 0.000052 and the post-QC Bonferroni threshold for our Discovery analysis p < 0.00015. Models used pairwise combinations of sample size N = 200 and β-coefficients (from β = 0 to β = 1, by 0.1). For each combination, the model was simulated 1000 times. Average power was determined as the proportion of p-values below the significance threshold. The dashed line indicates 80% power. The false positive rates are available in Supplemental Table 1. Comparing this figure to the previous Supplemental Figure 1, one sees that the trajectories of the power curves differ when considering a dichotomous versus continuous predictor. Quality control appeared to have a greater improvement on power when utilizing a binary rather than a continuous predictor. This reinforces the idea of the separation of variable types for QC, as categorical predictors may need more stringent thresholds (such as a larger sample size minimum) or additional steps (a minimum category size or case-control ratio filter).

**Supplemental Table 1. False positive rate across simulations.** Simulations used a continuous predictor or dichotomous predictor with proportions of exposure as described. Models used pairwise combinations of sample size N=200 and β-coefficients from β = 0 to β = 1. For each combination, the model was simulated 1000 times and the false positive rate was determined by the number of significant p-values returned when β = 0. None of the simulations performed garnered an inflated burden of false positives.

| Model | Predictor | QC-Status | Proportion Exposed | False Positive Rate |
| --- | --- | --- | --- | --- |
| 1 | Continuous | Pre-QC | -- | 0 |
| 2 | Continuous | Post-QC | -- | 0.001 |
| 3 | Binary | Pre-QC | 0.1 | 0 |
| 4 | Binary | Pre-QC | 0.2 | 0 |
| 5 | Binary | Pre-QC | 0.3 | 0 |
| 6 | Binary | Pre-QC | 0.4 | 0 |
| 7 | Binary | Pre-QC | 0.5 | 0 |
| 8 | Binary | Post-QC | 0.1 | 0 |
| 9 | Binary | Post-QC | 0.2 | 0 |
| 10 | Binary | Post-QC | 0.3 | 0 |
| 11 | Binary | Post-QC | 0.4 | 0 |
| 12 | Binary | Post-QC | 0.5 | 0 |

**Supplemental Figure 3.** A) shows a highly right-skewed distribution value of 1.131507 for BMI from the discovery dataset. B) shows a very low skew graph with a distribution value of 0.4123728 after log transforming the BMI values to normalize the data.


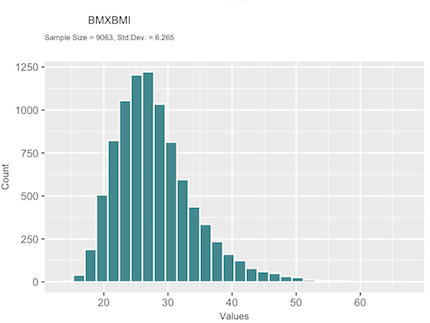

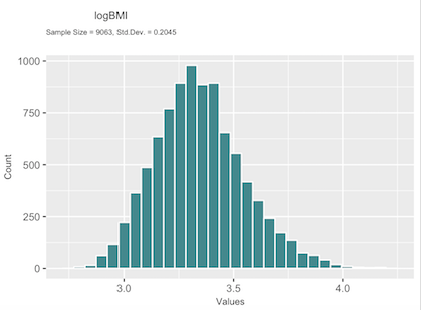


A

B

**Supplemental Figure 4.** Manhattan plots displaying categories of variables along the x-axis with log transformed p-values along the y-axis, adjusted exposures: T2D (A), CAD (B), HDL(C), LDL (D), TG (E), and TC (F). The discovery (circles) and replication (squares) results are compared while visualizing a Bonferroni p < 0.05 threshold.


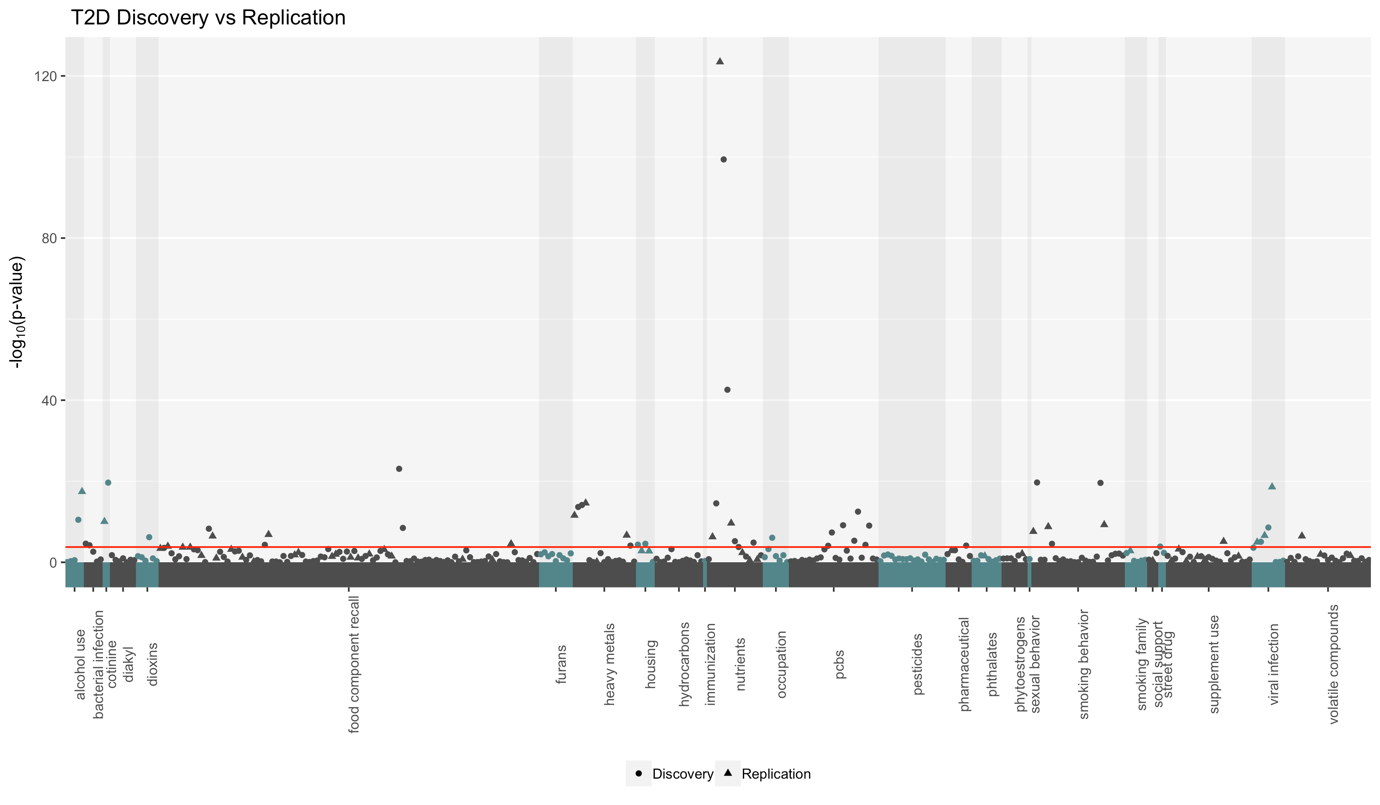


A

B


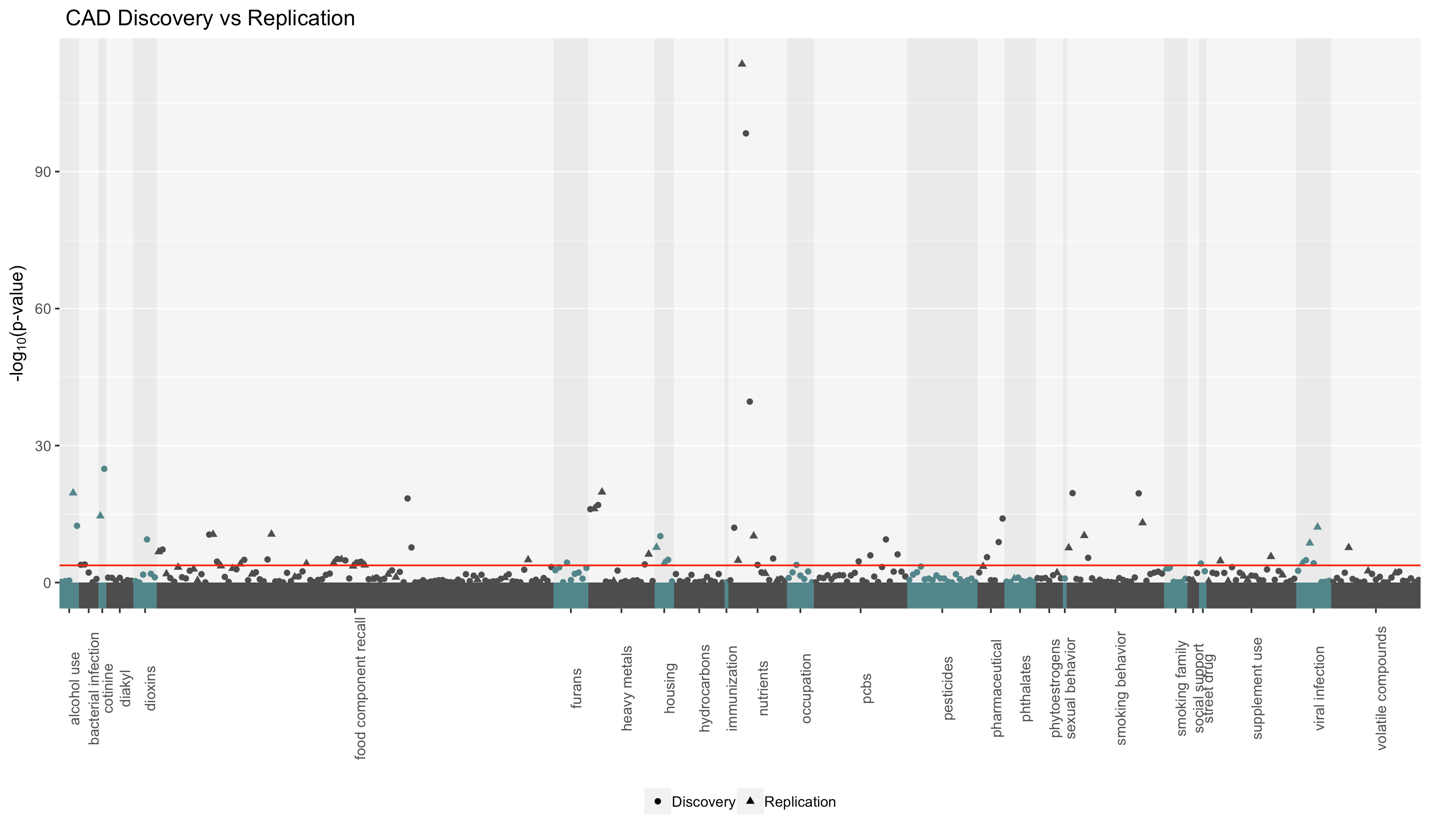


C

**
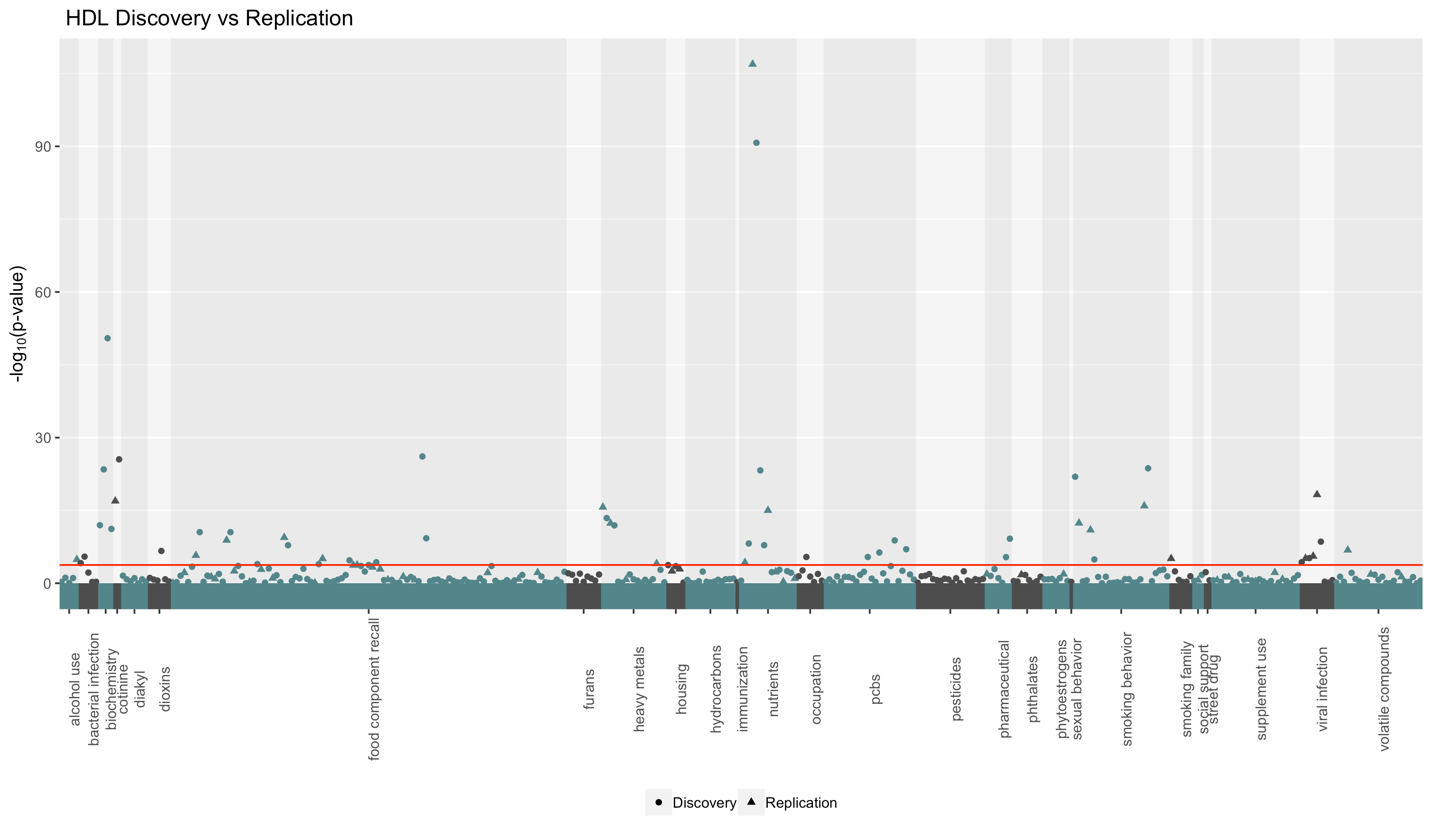
**

D


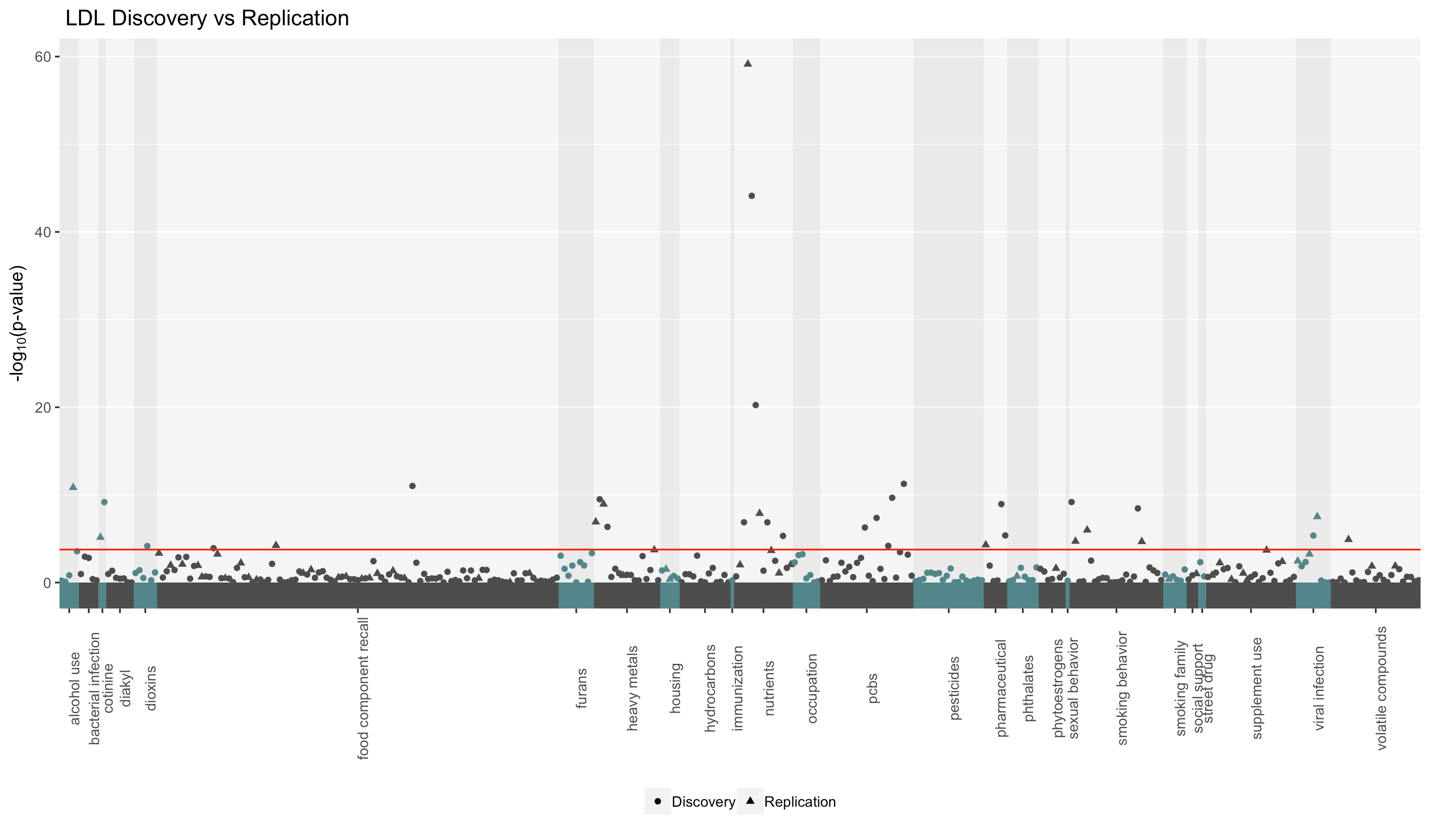


E

**
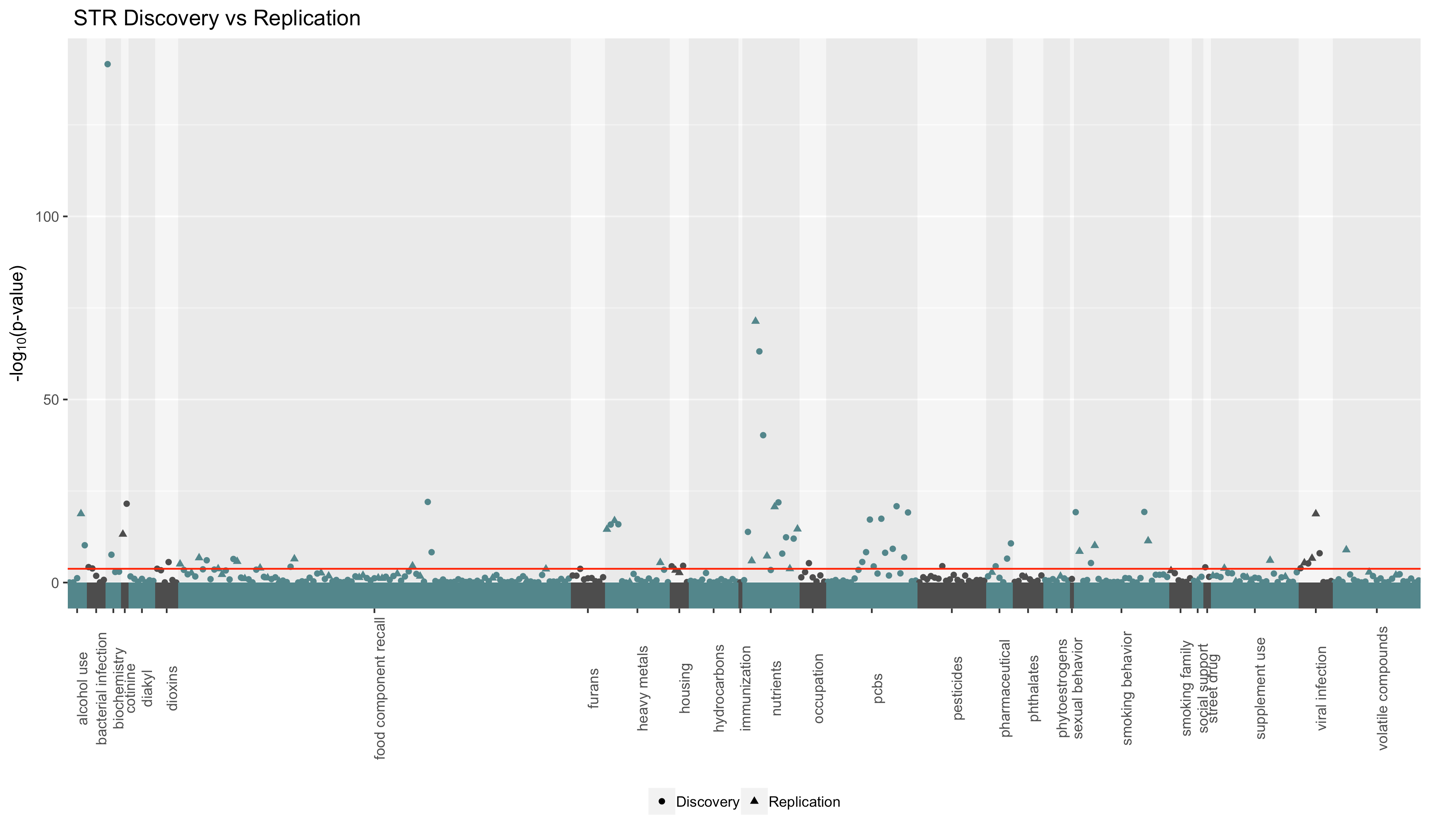
**

F

**
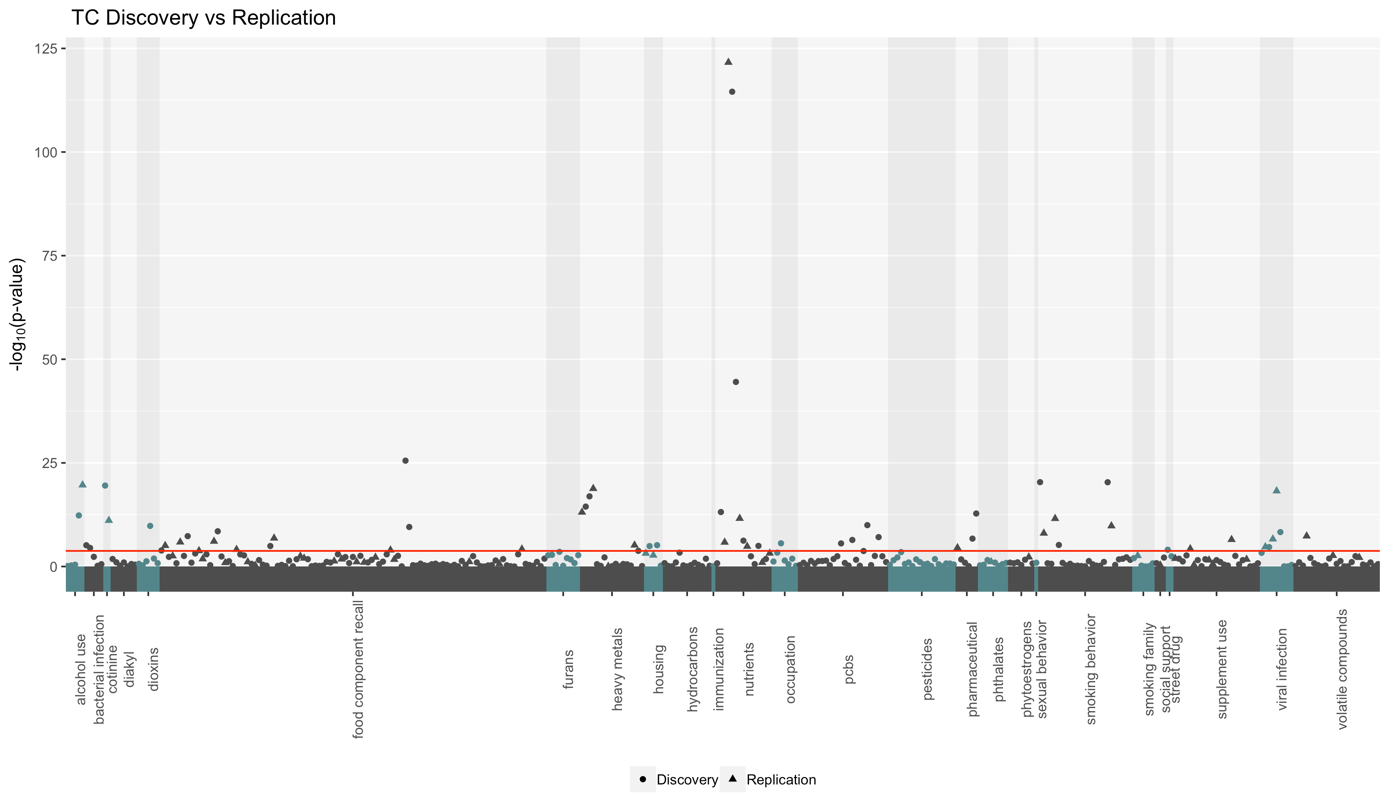
**

Reference

Hall, M. A., Moore, J. H., and Ritchie, M. D. (2016). Embracing Complex Associations in Common Traits: Critical Considerations for Precision Medicine. *Trends Genet.* 32, 470–484. doi:10.1016/j.tig.2016.06.001.
